# Smooth Sidewalls on Crystalline Gold through Facet-Selective Anisotropic Reactive Ion Etching: Toward Low-Loss Plasmonic Devices

**DOI:** 10.1021/acs.nanolett.1c04405

**Published:** 2022-06-02

**Authors:** Alexander B. Greenwood, Krishna C. Balram, Henkjan Gersen

**Affiliations:** † Nanophotonics and Nanophysics Group, H. H. Wills Physics Laboratory, University of Bristol, Bristol, BS8 1TL, United Kingdom; ‡ Quantum Engineering Technology Laboratories and Department of Electrical and Electronic Engineering, University of Bristol, Woodland Road, Bristol BS8 1UB, United Kingdom

**Keywords:** plasmonics, slot waveguides, etching, surface roughness, quantum emitters

## Abstract

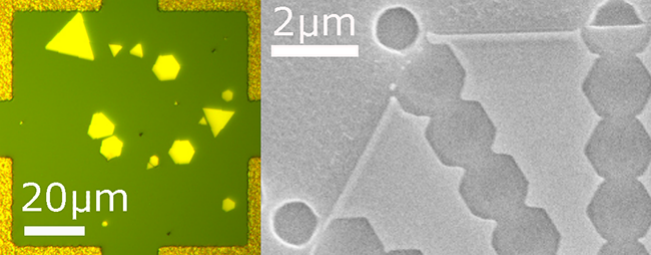

Quantum plasmonics aims to harness the deeply subwavelength confinement provided by plasmonic devices to engineer more efficient interfaces to quantum systems in particular single emitters. Realizing this vision is hampered by the roughness-induced scattering and loss inherent in most nanofabricated devices. In this work, we show evidence of a reactive ion etching process to selectively etch gold along select crystalline facets. Since the etch is facet selective, the sidewalls of fabricated devices are smoother than the lithography induced line-edge roughness with the prospect of achieving atomic smoothness by further optimization of the etch chemistry. This opens up a route toward fabricating integrated plasmonic circuits that can achieve loss metrics close to fundamental bounds.

Quantum plasmonics is an area of research at the intersection of plasmonics and quantum photonics that uses the confinement of electromagnetic fields to nanoscale dimensions intrinsic to surface plasmons to greatly enhance light–matter interactions.^1^ This exceptionally strong confinement would enable a quantum emitter to direct its emitted photons into a plasmonic mode with near unit probability.^2^ For this reason, hybrid systems consisting of quantum emitters coupled to plasmonic waveguides have received considerable attention as building blocks for future quantum plasmonic circuits.^3−5^ Interestingly, the electrical field strengths in plasmonic structures can be several orders larger compared to structures fabricated in dielectrics making it easier to induce nonlinear behavior.^6−9^ These nonlinearities can not only shift the phase of propagating plasmons needed in interferometers but also can induce plasmon–plasmon interactions, which takes place on picosecond time scales enabling new applications not feasible in all-dielectric photonic circuits.^10^

The ultimate vision in the field of quantum plasmonics is to build arbitrary complex plasmonic circuits in which plasmons can strongly interact with emitters and thereby enable classical and quantum information processing with a very small on-chip footprint.^11^ To achieve this, the development of advanced techniques for fabricating two-dimensional metallic nanostructures with high quality and well-defined geometries is key.^2^ Unfortunately, achieving this in practice is challenging as plasmonic structures fabricated with electron-beam lithography and thermally evaporated metals show strong plasmon propagation losses due to scattering induced by the polycrystalline nature of metals deposited in this manner^12^ in addition to the sidewall roughness induced by the lithography and reactive ion etching used for pattern transfer. Surface plasmon waves are particularly sensitive to such surface inhomogeneities as they exist very close to the interface.^13^

As we move from 1D confinement in surface plasmons to 2D confinement in metal insulator metal waveguide (MIM) geometries, this problem is further exacerbated.^14^ As the field confinement is increased, the proportional effect of surface/sidewall roughness on the propagating waveguide mode is magnified. This is shown in Figure 1a, where the effect of surface roughness on plasmonic slot (Figure 1b) and dielectric waveguides (Figure 1c) at λ = 650 nm is quantified by estimating the normalized change in mode index as a function of the perturbation size. This perturbation is denoted as a particle in Figure 1b,c. Given the tight modal confinement (slot width *w* = 75 nm, slot thickness *t* = 100 nm), even small scale roughness (∼5 nm) can have a significant effect on the propagating waveguide mode and lead to excess scattering and loss, as also found by Wang et. al.^14^ This has been one of the key reasons why plasmonic devices in practice have losses that far exceed what simulations predict, which typically only account for ohmic losses in the metal. Addressing nanofabrication-induced surface roughness loss is critical if quantum plasmonic circuits are to become a reality. While these calculations have been done for propagating modes, the ideas can be extended to localized modes in plasmonic nanocavities and bullseye antenna structures, commonly employed for enhancing light–matter interaction.

**Figure 1:**
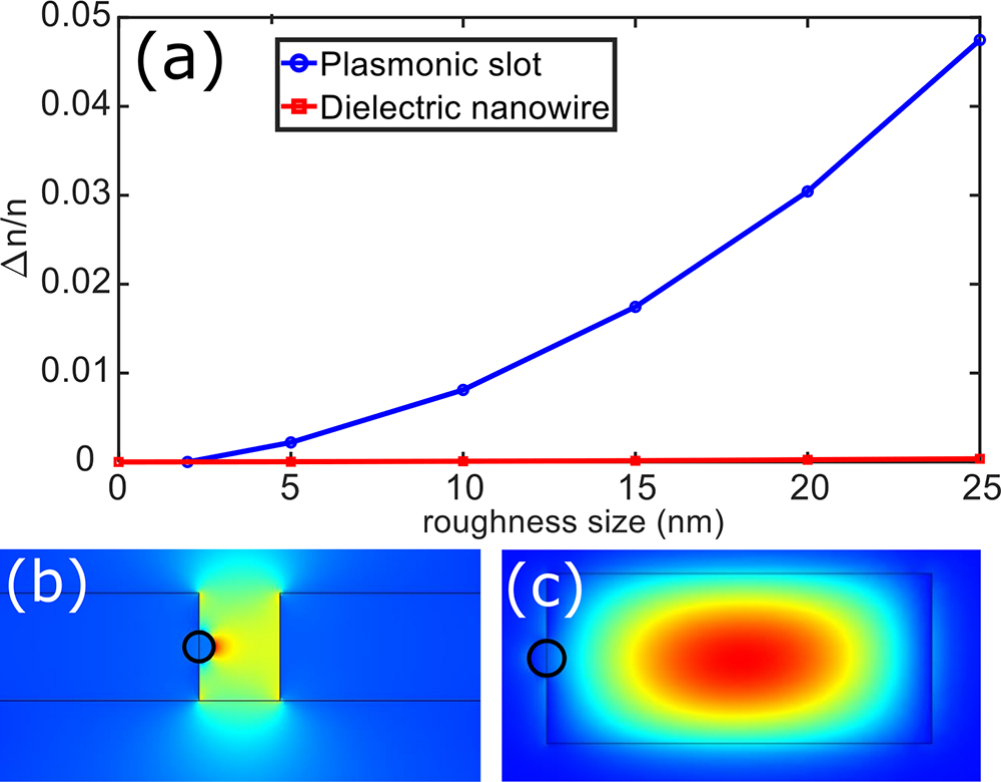
(a) Plot showing the normalized change in the waveguide mode index (Δ*n*/*n*) as a function of roughness size for dielectric (Si, *w* = 500 nm, *t* = 220 nm) and plasmonic slot waveguides (Au, *t* = 100 nm, *w* = 75 nm) at 650 nm. The roughness in these calculations is modeled by a single spherical particle at the center of the waveguide mode as shown in the field plots in (b,c). The unperturbed mode index is used to normalize the change in the two cases. As can be seen, the plasmonic slot waveguide is far more sensitive to surface roughness as compared to the dielectric waveguide.

While epitaxial growth of metal films can lead to atomically smooth crystalline films, there is still the issue of roughness induced by nanofabrication (both the lithography and pattern transfer). It is very challenging to reduce surface roughness of electron beam lithography below 5 nm, especially for resists with thickness >100 nm as used for most lift-off and pattern transfer approaches. The only way to achieve a sidewall roughness that is lower than the lithographic roughness is to develop an etch process that smooths the lithographic roughness. This can be achieved by having some chemical selectivity in the reactive ion etching process where the etch chemistry is preferential to certain atomic planes. This method is widely used in alkaline etching of silicon using potassium hydroxide (KOH) and tetra-methylammonium hydroxide (TMAH)^15,16^ to make atomically smooth devices for MEMS (inertial sensors) and optics (X-ray phase masks and gratings) but is generally thought to be infeasible with dry etching methods on noble metal single crystals.^17^ Here, we develop an anisotropic dry etching recipe that shows preferential etching of gold planes and sidewall roughness that is smoother than the underlying roughness of the lithography.

Strong mode confinement and large propagation lengths have been achieved with chemically synthesized metallic nanowires^18^ that do not suffer from fabrication induced roughness; however, these structures are hardly suitable for a controlled circuit design. Two dimensional colloidal gold crystalline flakes can be grown using chemical synthesis methods that are typically up to tens of micrometers in lateral dimensions,^19^ although examples of millimeter-sized crystals have been reported.^12,20,21^ Top-down lithography on such colloidal gold crystals would combine the best of both worlds,^22,23^ that is, well-defined single crystal two-dimensional gold flakes as the substrate in which a controlled geometry can be fabricated using simple and widespread electron-beam lithography and dry etching instrumentation.^24,25^ Fabrication methods such as focused ion beam have been used in the past,^26^ and the downside of such methods is the inherent contamination with gallium ions, reducing plasmonic efficiency. However, techniques using gold ions have also been explored.^27^ The need for better etch and pattern transfer techniques is perhaps best illustrated by recent work^28^ on building plasmonic circuits in silver crystals, where only 20% of the expected signal, from emitter waveguide coupling, was measured experimentally, and 80% was lost due to roughness-induced scattering after FIB patterning.

Here we show evidence of an anisotropic etch process combining widely utilized e-beam lithography and dry etching methods. This etching method holds promise for the ability to fabricate structures along crystal boundaries resulting in smooth sidewalls and without the contamination of gallium ions. Such an approach could greatly increase the efficiency of plasmonic structures, such as waveguides or interferometers.

The crystal growth method is similar to the one demonstrated by Wu et al.^19^ with the schematic layout depicted in Figure 2a. Before growth, a silicon substrate with fiducial markers is cleaned through a 10 min sonication with acetone, followed by a second 10 min sonication with isopropanol and dried with a nitrogen spray gun. The substrates are suspended vertically in 40 mL of ethylene glycol heated to 50 °C in a water bath. The ethylene glycol solution is stirred at 200 rpm and 360 μL of 0.1 M gold(III) chloride trihydrate in ethylene glycol is added. After 20 min, 360 μL of 0.1 M aniline within ethylene glycol is added and left for a further 5 min. The stirring is then stopped and kept in an enclosed water bath at 50 °C for 24 h. The substrates are removed and sequentially washed with methanol and IPA under sonication for a further 5 min each. The resulting crystals can be seen in Figure 2b as imaged by optical microscopy. The preferential growth of the crystal results in the {111} plane lying parallel to the image plane.^22^ It should be noted that the orientation of the substrate with respect to the rotation of the stirrer bar has an effect on the concentration of crystals on the surface, with the side facing the flow generally having a greater number of crystals. To be able to etch the desired structures into individual gold crystals, a mask covering the full sample is required to enable writing all structures in a single run in an e-beam lithography system. This requires not only the location of the crystals but also their orientation so that the crystal facet direction is known with respect to the written structure. Therefore, the substrate contains fiducial grid markers so that the location and orientation can be determined; this allows each crystal to be assigned a tailored structure. The array of labeled grid markers was produced on the sample through photolithographic means. These fiducial markers were initially formed through thermal evaporation of a Cr/Au layer; however, the gold layer was found to react during the crystal growth, causing a significant reduction in the number of suitably sized crystals. For that reason, all substrates containing fiducial markers were produced through etching of the silicon substrate thereby preventing any reaction between the markers and growth solution.

**Figure 2:**
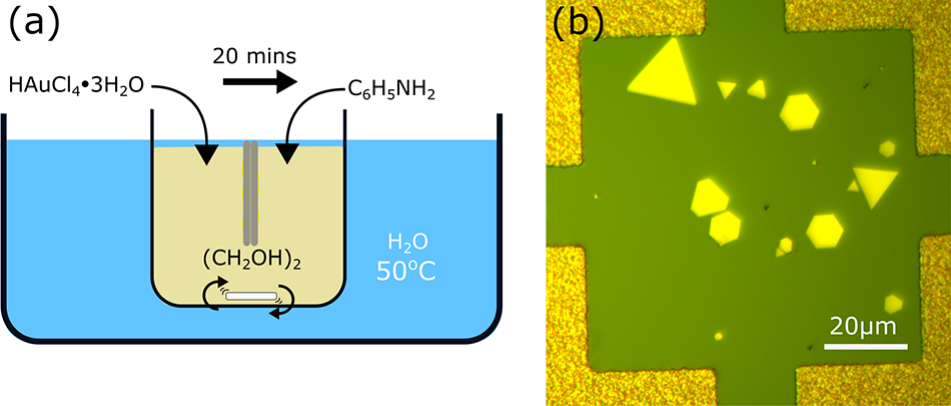
(a) Schematic layout for the crystal growth reaction. Two substrates are suspended in (CH_2_OH)_2_ heated in a water bath. While stirring, HAuCl_4_·3H_2_O is added. After 20 min, C_6_H_5_NH_2_ is added. (b) Optical image of the crystal structures grown on a silicon substrate with gold fiducial markers.

Following the growth stage the relative location and orientation of each gold crystal was identified with respect to the fiducial markers. The sample was subsequently coated in MaN, a negative e-beam resist, and exposed using a Raith Voyager e-beam lithography system. The sample was then etched using an inductively coupled plasma reactive ion etch (Oxford Instruments System 100 ICP 180) with the etch parameters stated in Table 1, based on the work by Aydemir and Akin.^29^ This method was chosen to minimize the amount of gold redeposition during the etching process to reduce surface roughness of the final structure. Here, inductively coupled plasma (ICP) power dictates the ion density whereas the radio frequency (RF) power is used to accelerate the ions toward the surface. A 10 mTorr spike is used for initial plasma generation and tuning of the coils before dropping to 5 mTorr for the duration of the etch process.

**Table 1:** ICP Etch Parameters for Etching of Gold Crystals

ICP power	600 W
RF power	250 W
argon flow rate	5 sccm
chlorine flow rate	15 sccm
pressure	5 mTorr (10 mTorr spike)
temperature	40 °C

An etch time of 60 s was found to be optimal in generating the test structures seen within this paper. Higher durations generally resulted in the complete removal of the mask due to the nonspecific etch characteristics of a Cl etch. However, we would like to stress that this is a first step in the direction of developing facet selective dry-etching of noble metals, and further work is needed to fully optimize the etch parameters Specific masks were created to test this etch recipe, a single 1 μm circular hole and zigzag lines, orientated both along the crystal facets and 30° from the facets. The results are shown in Figure 3. Figure 3a shows the result from an etch with a simple 1 μm circular hole mask. Figure 3b identifies this mask with a red line mask pattern. Rather than etching in a uniform circle corresponding to the mask, the crystal is etched quicker along set crystal facets, resulting in a hexagon shape. This is further confirmed by the resulting structure from the zigzag structure seen in Figure 3c with the mask in Figure 3d, where the higher detail zigzag structure that can also be seen in the mask residue in Figure 3c is lost when the line is oriented along one of the crystal faces. The lines orientated along crystal faces are much smoother and do not show evidence of the initial zigzag structure. Not all of the crystal in Figure 3c appears to be etched, this is suspected to be a result of the mask not being fully removed due to the close proximity of one of the fiducial markers. Failure to remove this mask not only protects the crystal from being etched but also causes the rough texturing that can be seen in the SEM image. Figure 3e further reveals the etching process. Here, a distinct height difference is seen along the surface of the structure, and it is believed that this is the remaining gold while the rest of the structure is silicon; this is thought to occur as a result of the gold being etched while directly under the mask. As seen in Figure 3f, the etch structure does not match the mask, preferentially following crystal boundaries and in fact, as identified by the arrows, does not always etch under the mask given the right conditions. These images show that just like there is a preferential growth direction of the crystals, there is a preferential etch direction when utilizing this etch method.

**Figure 3:**
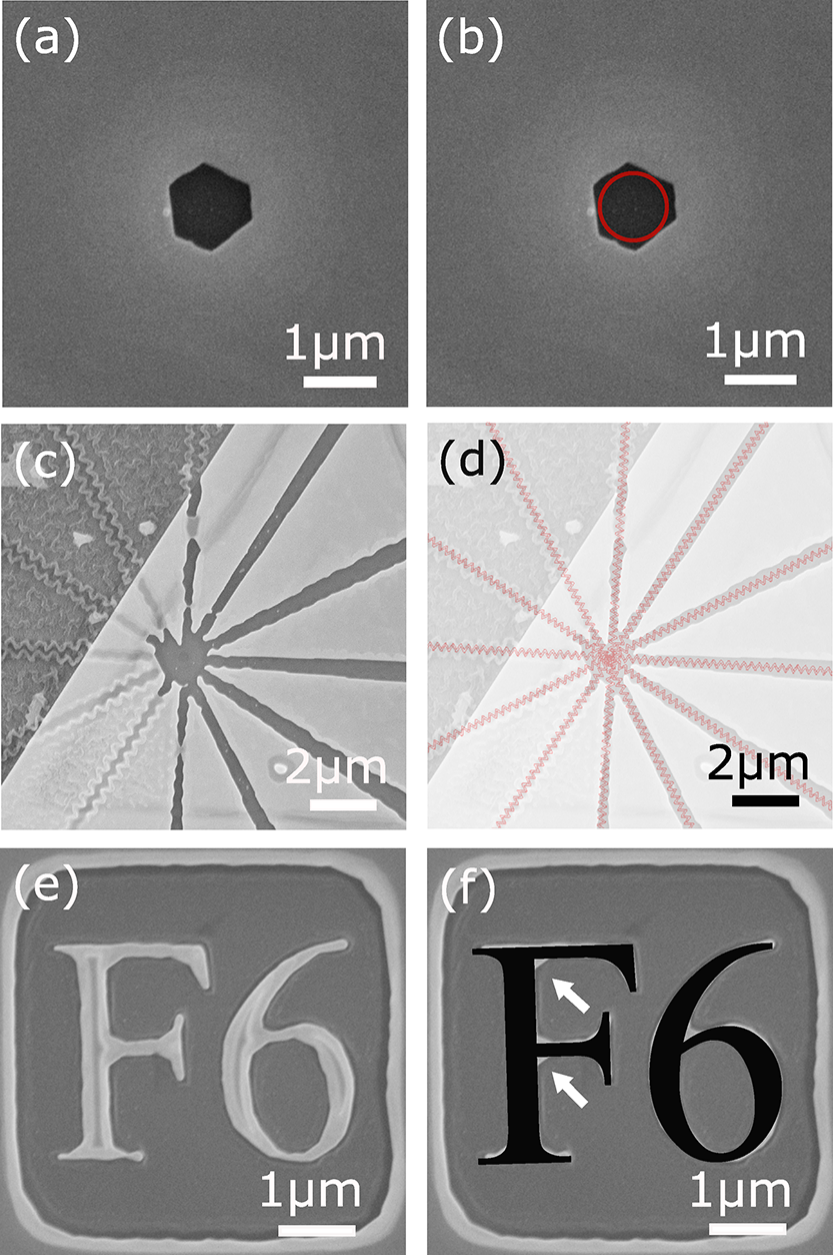
(a) Hole and (b) mask overlaid on SEM images of the etched structure, respectively. (c,d) SEM images of the resulting etch and the zigzag mask, respectively. (d) Unusual roughness shown in the top corner of the image along with a limited etch into the gold; this is believed to be caused by unremoved photoresist. (e) Resulting etch from a letter and number mask, seen in (f). Arrows in (f) highlighting deviation from the masked structure.

Although this appears to prevent etching plasmonic structures in colloidal gold crystals, this effect could in fact be exploited in unexpected ways when it comes to designing specific structures that have minimal loses. Figure 4a shows how different design considerations can create a variety of structures. By aligning a number of 1 μm holes along a crystal face, it can be seen how if left for longer etch time the resulting etch would have smooth side walls aligned with a crystal face, similar to etching methodologies used in silicon^16^

**Figure 4:**
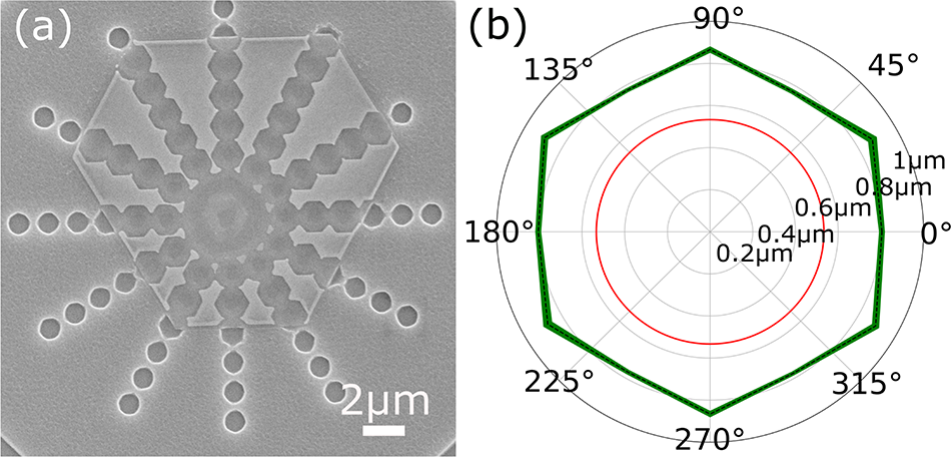
(a) Numerous 1 μm holes orientated along different angles with respect to the crystal faces. (b) Polar plot identifying the etch characteristics for a 60 s etch using the recipe in Table 1 through a sequence of 1 μm holes, seen in (a). Angles identify the direction etched with 0° at the top of the crystal seen in (a). The red shows the etched distance in silicon with the green denoting the crystal etch distance.

The characteristics of this in plane etch are displayed in the polar plot in Figure 4b showing the distance etched along various axis with the green line depicting etch distance for the crystalline gold and for comparison in red the resulting etch into the silicon substrate. The silicon etch was determined by averaging the diameter over a number of circle etch patterns, all from within a 30 μm range on a single sample. A similar method was used for determining the crystalline etch with distances taken along 0°, 30°, 60°, 90°, 120°, and 150°. The thickness of the line depicts the standard error for the etch distance measurements. Although there is minor etching below the mask within the silicon substrate, as depicted by the hole diameter slightly over 1 μm, there is a significant underetch for the gold crystal, as seen by the significantly larger hole in the crystal. There does appear to be some discrepancy between the etch rate seen in Figure 3a and Figure 4; this is not fully understood but is suspected to be a result of greater access for the etchant to the surface. This is backed up by seeing that in the center area all hole structures have been overetched, removing any evidence of their pattern, although ebeam-induced proximity effects potentially also play a role in this. We would like to note here that the facet selectivity (etch anisotropy) is lower than what is traditionally observed in chemical etchants and may limit the ultimate sidewall smoothness in our devices. One of the key future directions of this work is to map out the etch anisotropy more completely in terms of the etch parameter space and construct recipes that significantly improve on this anisotropy.

In the cases in which the holes in Figure 4a are not aligned correctly with the crystal faces, it is clearly seen that zigzag lines are formed, posing a problem for some structures. If, however, structures and devices are designed with this in mind and the etch process is correctly controlled, then it should be possible to create very smooth edges following the crystal faces.^16^ By designing the structure so that edges only lie along the crystal face direction, fabrication not only becomes possible but should also result in a smoother structures due to the preferential etch. Some example structures are shown conceptually in the right column of Figure 5a, where the geometry of the structures are confined to the crystal faces with their silicon counterparts shown in the left column. The SEM image in Figure 5b shows a fabricated hexagonal interferometer that follows the crystal boundaries. Because of a rapid underetch that occurs for the crystalline gold, the interferometer seen here is predominately silicon, as seen by the inset indicating the cross section of one of the interferometer arms (an AFM image providing additional evidence for this is supplied in the SI). Although the underetch in this particular test prevented the correct fabrication of the desired structure, with further study of the etch parameters and effect surrounding different masks this could be accounted for in future work. Although it is challenging to comment in detail on the actual sidewall smoothness obtained in our fabricated structures without performing high-resolution TEM images, our main goal is to demonstrate that plasmonic devices can be fabricated with lower surface roughness and can achieve higher performance (lower loss) than traditional FIB-based lithographic approaches.^28^

**Figure 5:**
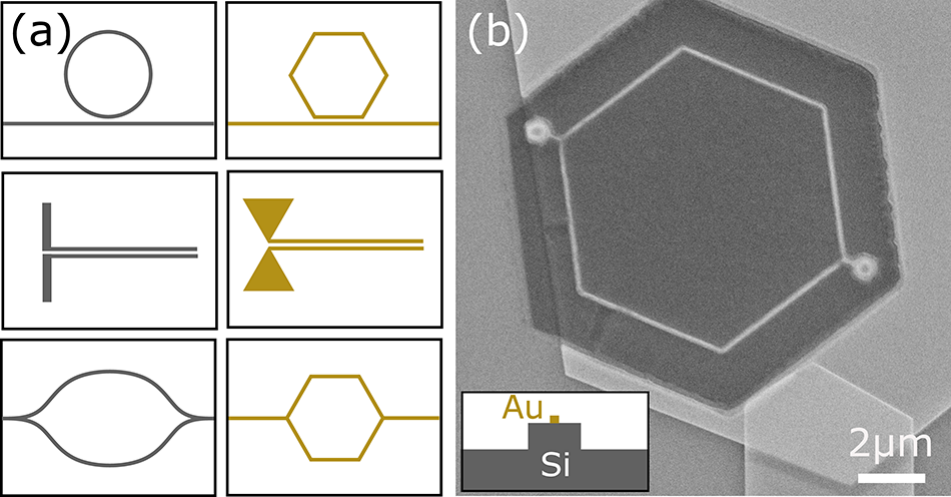
(a) Example structures (not to scale); left column converted to a crystalline design, right column to work alongside the crystalline etch. (b) SEM image of an example hexagonal interferometer structure following the crystal facets. The predominant material seen to make up this structure is silicon due to significant underetching of the gold leaving only a narrow section of gold on top.

Throughout this paper the fabrication of plasmonic devices from single crystalline materials using a chemical dry etching method was investigated. Our results show that dry etching by using ICP results in an anisotropic etch that could potentially enable the fabrication of atomically smooth side walls in crystalline plasmonic structures, provided that the designs for devices carefully consider the etching face.^15^ This formation of atomically smooth sidewalls could enable a significant reduction in loss for plasmonic devices fabricated in this way as plasmonics are particularly sensitive to surface inhomogeneities close to the surface.^13^ Further work to investigate the etch parameters and how they can be controlled to optimize the structures created following this process is currently planned.

## Supporting Information Available

AFM image of the structure seen in Figure 5b; additional underlying data supporting the paper (PDF)
