# Supplementary material for: Smooth Sidewalls on Crystalline Gold through Facet-Selective Anisotropic Reactive Ion Etching: Toward Low-Loss Plasmonic Devices
Source: Nano Lett. 2022 Jun 2;22(12):4617–21. doi: 10.1021/acs.nanolett.1c04405 (PMC9228404; doi:10.1021/acs.nanolett.1c04405)
Supplement: Supplementary file 1 — nl1c04405_si_001.pdf [file nl1c04405_si_001.pdf]

Supplementary information for: Smooth sidewalls on crystalline gold through facet-selective anisotropic reactive ion etching: towards low-loss plasmonic devices

Alexander B. Greenwood,[†] Krishna C. Balram,[‡] and Henkjan Gersen[*,†]

[†]*Nanophotonics and Nanophysics Group, H. H. Wills Physics Laboratory, University of Bristol, Bristol, BS8 1TL, United Kingdom*

[‡]*Quantum Engineering Technology Labs and Department of Electrical and Electronic Engineering, University of Bristol, Woodland Road, Bristol BS8 1UB, United Kingdom*

E-mail: h.gersen@bristol.ac.uk

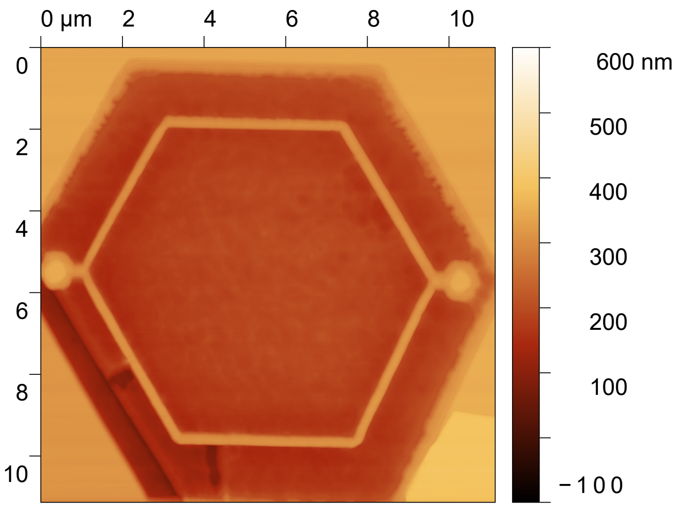

Figure 1: Atomic force microscope (AFM) image corresponding to the structure shown in Fig. 5(b) in the main text.

Atomic force microscope (AFM) image corresponding to the structure shown in Fig. 5(b) in the main text. The topographic information shows that the intended waveguide in Fig. 5(b) has a height that is lower than the surrounding crystalline flake, demonstrating the sample was over-etched. This can not easily be seen from the included SEM image. The scale bar used for this image also highlights that the process will etch into the underlying silicon substrate for areas that are not protected.
